# Hypertrophic pulmonary osteoarthropathy secondary to bronchial adenocarcinoma and coexisting pulmonary tuberculosis: a case report

**DOI:** 10.1186/1757-1626-1-221

**Published:** 2008-10-07

**Authors:** George Ntaios, Alexandra Adamidou, Dimitrios Karamitsos

**Affiliations:** 1 First Propedeutic Department of Internal Medicine, AHEPA Hospital, Aristotle University, Thessaloniki, Greece

## Abstract

A 44-year-old man presented with painful swelling of wrists and ankles, severe pain at both tibiae, clubbing of fingers and toes and arthritis in wrist and ankle joints. The chest roentgenogram showed consolidation of the right lower lobe, whereas plain roentgenograms revealed solid periosteal reaction at both tibiae. CT and bronchoscopy confirmed the presence of adenocarcinoma of the right lower lobe. Moreover, mycobacterium of tuberculosis was isolated by culture of the patient's sputum.

Our patient received antituberculous treatment and soon he underwent surgical excision of the tumour and subsequent chemotherapy. Ten months later, he returned with metastatic lesions in the brain and the adrenals. A few days later, he died.

The patient suffered from bronchial adenocarcinoma as well as pulmonary tuberculosis. As a complication of these two coexisting conditions, the patient developed hypertrophic pulmonary osteoarthropathy.

## Introduction

Hypertrophic pulmonary osteoarthropathy (HPOA) or else Pierre-Marie-Bamberger syndrome, constitutes a clinical syndrome characterized by clubbing of fingers and toes, arthritis and periostosis of the long distal bones. The lesions are usually bilateral and symmetric causing swelling and extreme pain of the affected limbs. It may occur as a primary condition, which is familial and affects mainly males [1]. However, in most instances, it occurs secondarily to conditions characterized by arteriovenous shunt like lung carcinoma, mesothelioma, pulmonary tuberculosis, congenital cyanotic heart disease, hepatic and colorectal carcinoma, inflammatory bowel disease, cirrhosis, pulmonary fibrosis and empyema [2,3]. The coexistence of two different conditions which both lead to hypertrophic osteoarthropathy is very rare. In this case report, we present the case of a 44-year-old man who developed hypertrophic osteoarthropathy due to bronchial carcinoma and coexisting pulmonary tuberculosis.

## Case report

**Figure 1:**
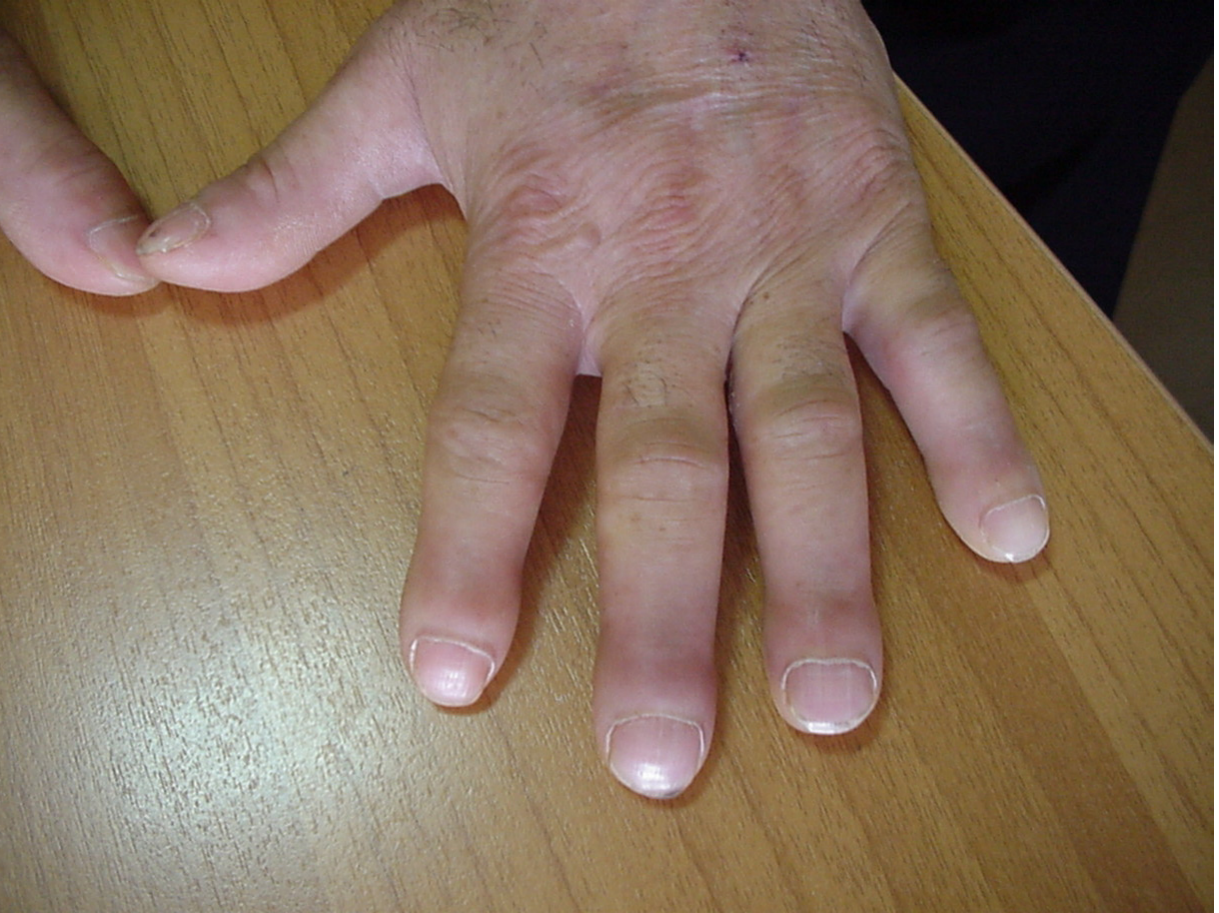
Finger clubbing.

A 44-year-old man was admitted in our clinic due to high fever and painful swelling of wrists and ankles for four months prior to admission. He also complained of severe pain at his tibiae. He was a heavy smoker (50 pack-years) and he denied any chronic illness. At admission, clinical examination revealed a severely ill patient who was unable to walk due to extreme leg pain. He was febrile (39°C) and arthritis of wrists and ankles could be easily noticed. Moreover, clubbing of the fingers was evident (figure 1).

Erythrocyte sedimentation rate and C-reactive protein were significantly increased (65 mm/hr and 8.6 mg/dl respectively) but leukocyte count was within normal values. The chest roentgenogram revealed consolidation of the right lower lobe. He was initially treated with broad spectrum antibiotics for three days without any clinical improvement. Meanwhile, computer tomography of the chest was performed, which revealed a solitary mass in the right lower lobe accompanied by a single mediastinal lymph node. There was no pleural effusion. Soon, bronchoscopy was performed which confirmed the presence of adenocarcinoma of the right lower lobe. In the meantime, mycobacterium of tuberculosis was isolated by sputum culture. Hence, our patient suffered from bronchial adenocarcinoma as well as pulmonary tuberculosis. As a complication of these two coexisting conditions, he developed hypertrophic pulmonary osteoarthropathy. Indeed, plain roentgenograms revealed a solid reaction of the periosteum of both tibiae. These findings were further confirmed by bone scintigraphy.

Our patient received antituberculous treatment and soon he underwent surgical excision of the tumour and subsequent chemotherapy. Ten months later, he returned with metastatic lesions of the brain and adrenals. A few days later, he died.

## Discussion

Coexistence of tuberculosis and lung cancer is fairly rare. However, there are different estimates about the prevalence of this combination. Watanabe *et al *suggested that it reaches 2.1% of patients with lung cancer [4]. This result was further confirmed by Cicėnas and Vencevičius [5]. However, Dacosta and Kinare identified this combination in 13.1% of patients with lung cancer [6].

Cicėnas and Vencevičius also indicated that epidermoid carcinoma is the lung tumour which is mainly associated with tuberculosis [5]. On the contrary, bronchial adenocarcinoma, which was the case in our patient, was diagnosed only in 21.7% of cases.

Although the association of HPOA with bronchial carcinoma and pulmonary tuberculosis is well known, the exact pathogenetic mechanism of the syndrome is still unclear. It was suggested that in cases of lung diseases, congenital cyanotic heart diseases and liver cirrhosis, megakaryocytes bypass the pulmonary circulation through arteriovenous shunting and escape fragmentation. Then, these unfragmented megakaryocytes stimulate the production of platelet derived growth factor (PDGF) and vascular endothelial growth factor (VEGF) by endothelial cells, resulting in angiogenesis and endothelial hyperplasia which is manifested as clubbing of fingers and toes [7].

Summarizing, we present a rare case of HPOA due to coexistence of bronchial adenocarcinoma and pulmonary tuberculosis. The presence of a readily recognised cause of HPOA should not prevent the clinical doctor from excluding other potential coexisting pathologic conditions that could also contribute to HPOA.

## Consent

Written informed consent was obtained from the patient for publication of this case report and any accompanying images. A copy of the written consent is available for review by the Editor-in-Chief of the ≪Cases Journal≫.

## Competing interests

The authors declare that they have no competing interests.

## Authors' contributions

GN, AA and DK were the treating physicians. GN drafted the manuscript. All authors read and approved the final manuscript.
